# Measuring Dynamic Leg Length during Normal Gait

**DOI:** 10.3390/s18124191

**Published:** 2018-11-29

**Authors:** Sam Khamis, Shmuel Springer, Dror Ovadia, Sima Krimus, Eli Carmeli

**Affiliations:** 1 Gait and Motion Analysis Laboratory, Department of Pediatric Orthopaedics, Dana Children’s Hospital, Tel Aviv Sourasky Medical Center, Tel Aviv 69978, Israel; khamisam@gmail.com (S.K.); simakrim@gmail.com (S.K.); 2 Department of Physical Therapy, Faculty of Social Welfare and Health Sciences, University of Haifa, Haifa 3498838, Israel; ecarmeli@univ.haifa.ac.il; 3 Physical Therapy Department, Faculty of Health Sciences, Ariel University, Ariel 40700, Israel; 4 Department of Pediatric Orthopedics, Dana Children’s Hospital, Tel Aviv Medical Center, Tel Aviv 69978, Israel; dovadia@tasmc.health.gov.il

**Keywords:** gait analysis, leg length discrepancy, measurement

## Abstract

Dynamic leg length [DLL] is a resultant factor of anatomic leg length and lower limb movement that is measured by the distance from the hip to the heel, ankle, and forefoot during the gait cycle. The aim of this study was to present DLL measurement during normal gait. Forty healthy participants underwent a gait evaluation using a motion analysis system. The average DLLs were compared between sides during the gait cycle using the paired *t*-test at 51 sample points. Time of maximal and minimal DLLs and the ratio between maximal and minimal DLLs during the gait cycle were calculated. DLLs were found to be consistent, indicated by a within standard deviation of <6.65 mm and by being symmetrical with no significant differences between sides [*p* > 0.103]. DLL patterns and time of maximal and minimal DLLs were established. The ratio between maximal DLLs during the stance phase and minimal DLLs during the swing phase was also defined and found to be symmetrical. Normative data of DLL measures were set with respect to magnitude and pattern during the gait cycle. These data might serve as a reference for abnormal gait deviation reflected by abnormal DLLs, thus promoting a new perspective in gait analysis.

## 1. Introduction

Leg length discrepancy (LLD) is a significant factor influencing several pathological and physiological conditions such as foot pathologies [1,2], low back pain [3,4], functional scoliosis in children [5], osteoarthritis of the hip and knee [6], impaired functional outcomes, and patient satisfaction after total hip replacement [7,8]. True leg length has been defined as an anatomic bony leg length [9,10,11], as opposed to a functional leg length incorporating true bony leg length, structural deformity, and joint position evaluated in a standing position [9,10,12]. 

Various imaging techniques have been used to measure anatomic leg length [6,13]. Radiography is considered the gold standard, with accepted methods such as full limb radiographs, scanograms, computerized tomography, and computerized digital radiographs [14,15]. These methods are highly reliable and valid; nonetheless, they are also expensive, not feasible for everyone, and expose the subject to radiation, thus impractical to use in routine clinical settings. In addition, radiography cannot measure functional leg length or detect functional leg length discrepancy dynamically. 

Two common clinical methods, the direct and indirect methods, are presently being used to measure LLD. The direct method measures the distance between the anterior superior iliac spine (ASIS) and the medial malleolus (MM), whereas the indirect method measures the differences in leg length between the sides while standing and uses lifts to level the pelvis [16]. The height of the lifts necessary to level the pelvis is equal to the difference in leg length. There is disagreement in the literature as to the reliability and validity of these clinical methods [17,18]. Their inability to measure all components involving leg lengths such as joint contractures, static or dynamic mechanical axial malalignment due to structural deformities, muscle weakness, or shortening, all of which are reflected in gait deviations, has recently led to a proposal of a new concept for measuring dynamic leg length [DLL] during the gait cycle [19]. 

**Figure 1:**
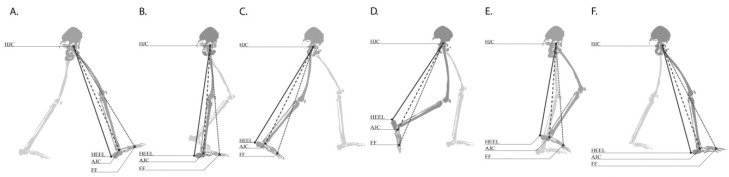
DLLs during the gait cycle phases measured from HJC to the HEEL, to the AJC, and to the FF. (**A**) Initial contact, (**B**) mid stance, (**C**) pre-swing, (**D**) initial swing, (**E**) mid swing, (**F**) terminal swing.

DLL is defined as the effective length of the lower limb, taking into account bony length, structural deformity, and kinematic angles of the lower extremity in the sagittal, frontal, and horizontal plane measured during the gait cycle [19]. It measures functional leg length during gait and detects functional leg length discrepancies due to structural and dynamic deviations [19,20,21]. DLLs are measured from the hip joint center (HJC), to the heel (HEEL), ankle joint center (AJC), and to the forefoot (FF), (Figure 1) [19].

DLL was found asymmetrical in two subjects presenting with true LLD and abnormal clinical findings leading to asymmetrical gait deviations [20], and in simulated LLD conditions [21]. 

LLD is usually detected by the presence of one or several abnormal kinematics parameters when assessing gait [22,23]. Kinematic strategies are employed to functionally lengthen the shorter limb and simultaneously shorten the longer limb [24,25,26,27]. These kinematics variations might be small or non-significant at each joint; however, their total effect might lead to a significant effect on the DLL [21]. Therefore, a dynamic measurement is required to assess the changes in the functional leg length of the lower limbs during gait. Understanding and early detection of the presence of a DLL discrepancy can enhance treatment. If caused by functional abnormalities such as muscle-tendon shortening or soft tissue lengthening, these should be directed accordingly. However, if they are caused by muscle weakness, then muscle strengthening will be targeted. If initiated by true LLD, then lift intervention [28] should be considered. 

The goal of this study was to present DLL measurement of healthy individuals performed by quantifying functional leg length changes during the gait cycle and investigate the biomechanical change in DLL. If applicable, this method may assist clinicians in gait laboratories in detecting and quantifying DLLs during pathological gait by comparing them to normative values. 

## 2. Materials and Methods

Forty healthy participants (20 women, 20 men), mean aged 26 years (19–37), with mean weight 64.5 kg (55–92), and mean height 170 cm (158–187), participated in the study. Inclusion criteria included no history of musculoskeletal injury or pain during the last year and no structural LLD > 5 mm. Structural LLD was assessed in a supine position by measuring the bilateral distance between the ASIS to the ipsilateral MM, using a tape measure. The mean of the two readings was considered the correct reading. In addition, a thorough clinical and musculoskeletal assessment was performed to exclude any musculoskeletal disorders. The study was approved by the Medical Center’s Ethics (Helsinki) Committee.

Participants underwent a gait laboratory evaluation using a motion analysis system (Vicon^®^, Oxford Metrics, Oxford, UK), according to the PlugInGait model (PGM) [14], to measure gait deviations in the lower extremities and pelvis. Thirteen reflective passive skin markers were placed on the subject’s pelvis and lower limbs according to the PGM protocol. Three retro-reflective markers were used to spatially define the pelvis, thigh, shank, and foot. Joint centers were calculated according to the marker placement and the subject’s anthropometric parameters. Participants walked at their self-selected speed. Three random trials were chosen for further analysis, sampled from six captured trials, each trial consisting of two gait cycles for the left and right side.

### 2.1. Data Reduction and Analysis

DLLs were measured from HJC, to the HEEL, AJC, and to the FF (Figure 1) [19]. 

This distance is the functional length of the lower extremity and a resultant factor of true bone leg length and lower limb movement during the gait cycle [19]. HJCs and AJCs were determined by the PGM. HEEL and FF markers were placed along the foot axis. The FF marker was placed on the dorsal surface of the forefoot over the second metatarsal head according to the PGM protocol [29]. HEEL markers were placed on the back of the heel where the line connecting it to the FF marker reflected the long axis of the foot at the same height as the FF marker. A custom foot alignment device with a cross-hair laser, based on the novel device described by Wervey and Schwartz, was used to ensure proper placement of the lateral malleolus, heel, and forefoot markers [30]. The virtual trajectories of the HJCs and AJCs and the real marker trajectories of the HEELs and FFs were used to measure the absolute distances from the HJC to the HEEL (HJC-HEEL), to the AJC (HJC-AJC), and to the FF (HJC-FF) throughout the gait cycle [15]. HEEL and FF trajectories were used to measure the leg length throughout the stance phase when the foot contacted the walking surface; the HJC-HEEL during initial contact to the foot flat phase and the HJC-FF during the heel rise phase (Figure 1). During the swing phase, these trajectories were used to measure the most distant point closest to the walking surface; HJC-FF during the initial to the late mid-swing phase and HJC-HEEL during the terminal swing phase (Figure 1). The three DLLs were chosen as they can present different results since they are dependent on gait deviations in respect of the amount of angular changes and timing during the gait cycle. The DLL components analyzed were: (1) Difference in DLLs between sides throughout the gait cycle. (2) Time of maximal and minimal DLLs during the gait cycle. (3) The ratio between maximal DLLs measured during the stance phase and minimal DLLs measured during the swing phase.

By evaluating DLLs throughout the gait cycle, an assessment of differences and detection time of significant differences between sides was attained. Mid to terminal stance was defined as the stance phase; mid-swing was defined as the swing phase. The maximal stance and minimal swing phase DLL values were chosen due to the mechanical requirements of gait. These values would be most affected when the maximal functional leg length during stance is required to clear the contralateral side and the minimal swing phase length is required to clear the foot off the ground. During gait, kinematics strategies are used to influence change in functional leg length occurring at those phases of the gait cycle [24,25,26,27]. Kinematic strategies are used to functionally lengthen the lower limb during mid-stance phase while opposite kinematic strategy is used to shorten the lower limb during mid-swing phase [24,25,26,27]. Initial contact and foot off were determined using the vertical ground reaction force. Loading response was defined as 0–10% of the gait cycle; mid-stance was defined as 10–30%, and terminal stance as 30–50%. [31]. 

### 2.2. Statistical Analysis

DLLs were measured, graphically plotted and checked for variability. The average DLLs were compared between sides for differences using the paired *t*-test at 51 sample points during the gait cycle. *p*-values were corrected by the Benjamin-Hochberg (BH) procedure, thus guaranteeing a false discovery rate (FDR) control of 0.05. Time of maximal and minimal DLLs was also presented together with the ratio between maximal and minimal DLLs during the gait cycle. We have calculated the DLLs normalized to the physical measurement of leg length, as measured in a supine position from the ASIS to the MM. 

## 3. Results

**Figure 2:**
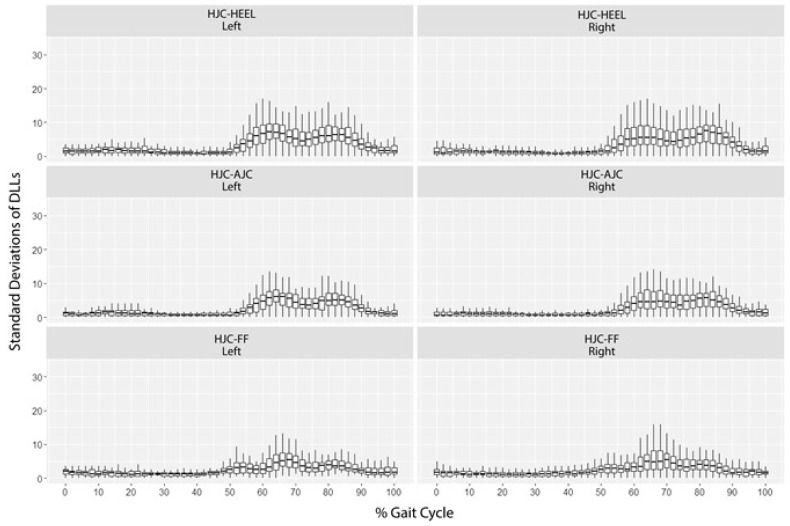
The within-subject DLLs standard deviation at each sample point during the gait cycle.

A systematic and consistent change occurred in all three DLLs. In 90% of the repeated measurements, the within-subject standard deviation at each sample point during the gait cycle was <6.65 mm, with a median of 1.73 mm (Figure 2). 

**Figure 3:**
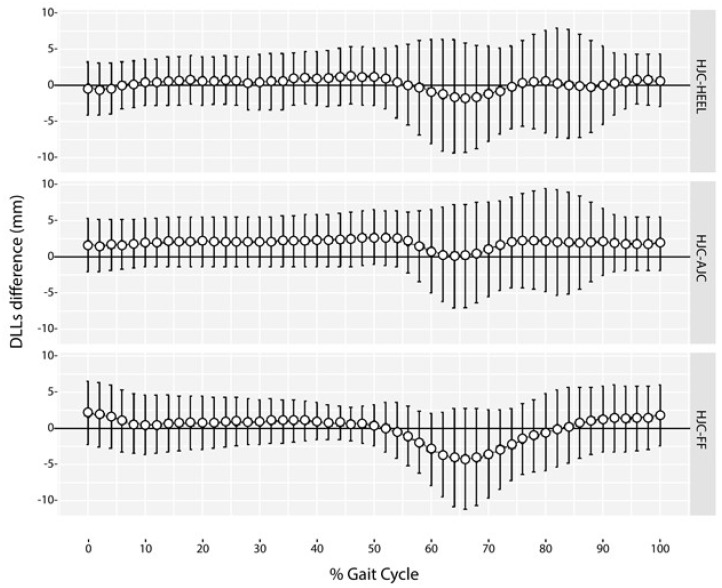
Adjusted 95% confidence intervals for mean average DLLs differences.

No significant differences in DLLs were found between sides throughout the gait cycle (minimal adjusted *p*-value > 0.103) (Figure 3).

**Figure 4:**
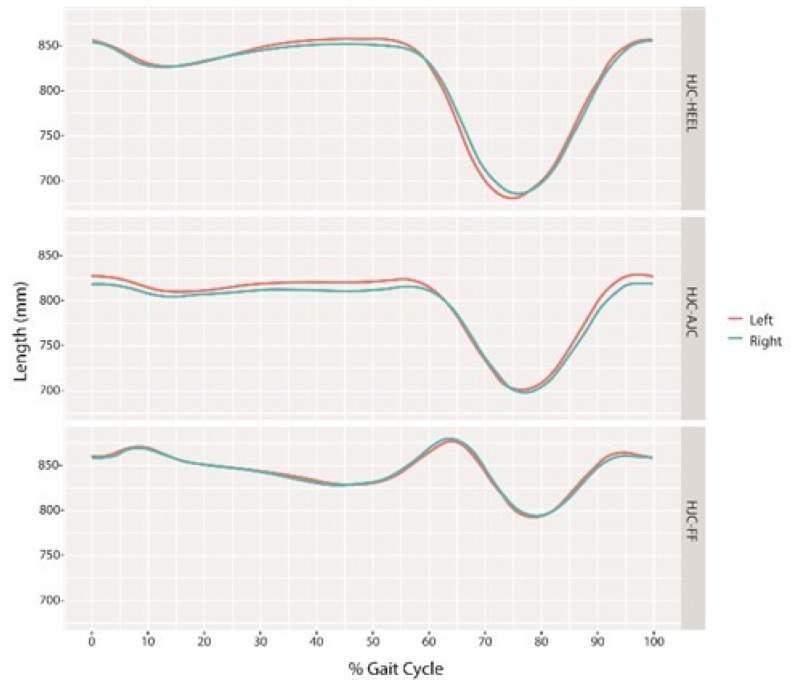
Average and standard deviation DLLs of one sampled subject as measured from the HJC to the HEEL (HJC-HEEL), to the AJC (HJC-AJC), and to the forefoot (HJC-FF).

DLLs were longest during the stance phase and shortest during the swing phase. Figure 4 characterizes the DLLs of one sampled subject. The HJC-FF demonstrated a slightly different pattern, whereby a gradual decrease in the HJC-FF occurred during the mid to terminal stance phase (10–50%) and was longest during the loading response (5–10%), pre-swing (50–60%), and initial swing phases (60–64%). 

**Figure 5:**
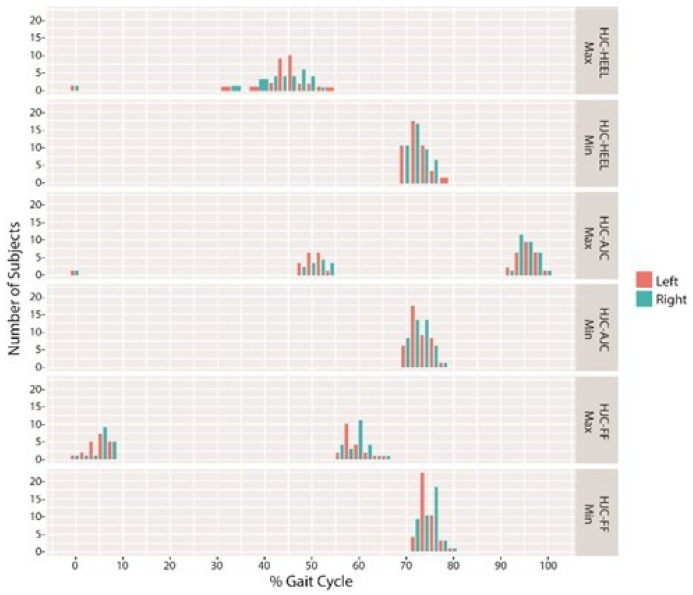
Time of maximal and minimal DLLs during the gait cycle.

As for the timing of maximal and minimal DLLs, the distance from the HJC to the HEEL and to the AJC was longest during the terminal-stance to the pre-swing phase (HJC-HEEL 32–54%, HJC-AJC 48–54%) and during the terminal swing to the initial contact phase (HJC-HEEL 94–100%, HJC-AJC 92–100%), (Figure 5).

**Figure 6:**
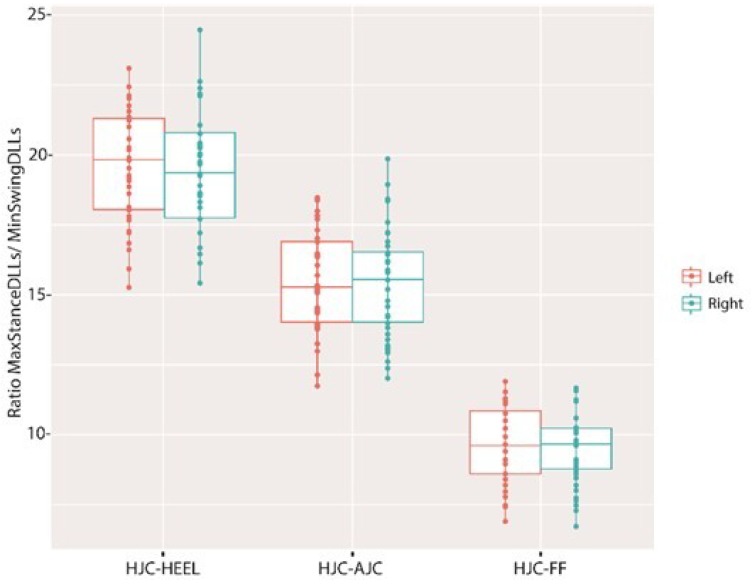
Ratio between maximal DLLs during the stance phase and minimal DLLs during the swing phase.

**Figure 7:**
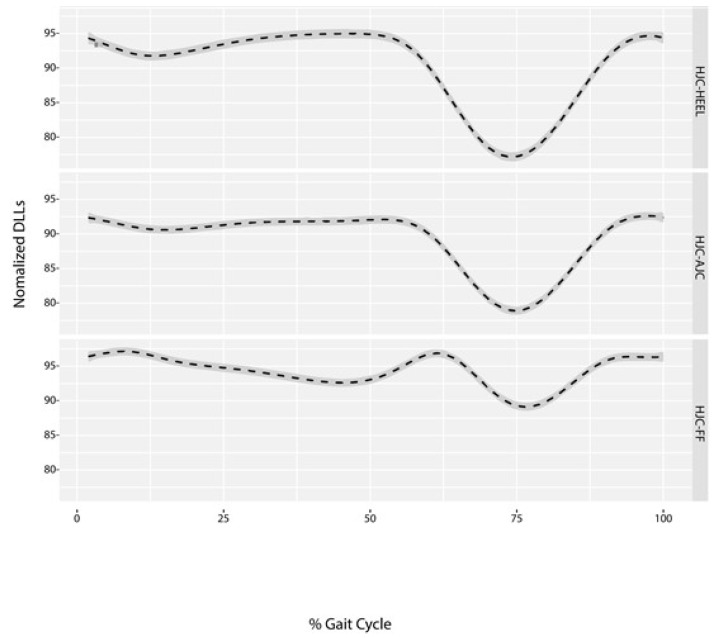
Normalized DLLs to leg length. Mean value (dotted line) ± standard deviation (grey area).

The distance was shortest during the initial to the mid-swing phase (70–78%) for both the HJC-HEEL and HJC-AJC. The HJC to the FF distance was longest during the loading response (2–8%) and during the pre-swing to the initial swing phase (56–66%) and shortest during the mid-swing phase (72–80%) (Figure 5). The ratio between minimal DLLs measured during the swing phase to the maximal DLLs measured during stance phase was 19% for HJC-HEEL, 15% for HJC-AJC, and 9% for HJC-FF, (Figure 6). The ratio was found to be symmetrical. DLLs were also normalized to the leg length measured in a supine position (ASIS to MM) (Figure 7).

## 4. Discussion

Our objective was to measure leg length during a normative gait cycle based on marker positions and center of joint location, relying on a valid clinical gait model. This feasible method quantifies the change in DLLs and assists in evaluating the mechanical changes in lower limb length during gait. The normative data that was acquired can be used as a reference for abnormal gait. All three DLL parameters (HJC to HEEL, AJC, and FF) were found consistent and with a non-significant difference between sides, thus implying symmetricity among healthy participants. The DLLs elongated during the stance phase and shortened during the swing phase. The gait cycle required a maximal length during the stance phase in order to clear the contralateral limb which must be at minimal length during the swing phase. Maximal HJC-HEEL and HJC-AJC lengths were found during the terminal stance to pre-swing phase allowing contralateral clearance. However, it was also longest during the terminal swing to the initial contact phase which appeared to be due to the mechanics of reaching forward to the next initial contact phase. HJC to FF was longest during the loading response and pre-swing to the initial swing phase due to its dependency on ankle plantar flexion movement, where maximal plantar flexion normally appears during the gait cycle [32] leading to HJC-FF elongation [20]. All DLLs measurements were found minimal during the mid-swing phase, thus allowing clearance of the lower limb. 

Normal timing of the functional change in leg length during the gait cycle is an important factor in achieving proper mechanics during walking and relying on proper timing of joint kinematics [27,32]. Joint kinematics compensating for LLD have been found to occur both during mid-stance and mid-swing in order to achieve dynamic lengthening or shortening of the lower limb at defined portions of the gait cycle [24,25,26,27]. Normal timing of maximal and minimal DLL was set as normal data which can be used as a reference for abnormal gait. Time of maximal DLLs was found to be more variable than minimal DLLs, however, it was confounded to two defined phases during the gait cycle, which can be attributed to the normal variability of kinematics of healthy subjects during gait [33]. A change in timing in leg length can indicate kinematic abnormalities, as was previously reported [20,34]. Accordingly, it may be possible to detect limitations during the gait cycle, preventing a normal change in the magnitude and timing of DLLs in pathological gait. 

An additional calculation was performed in an attempt to examine the normal changes in DLLs. Alterations in the DLLs were evaluated by measuring the change in length during the gait cycle. The ratio between the maximal stance phase length and the minimal swing phase length was calculated, finding a normative and symmetrical value when defining the percentage of decrease in DLLs during the gait cycle. The HJC-HEEL decreased by 19%, the HJC-AJC by 15%, and the HJC-FF by 9%. A higher ratio was due to a higher change in DLL caused by shortening during the swing phase. HJC-HEEL and HJC-AJC shortened during the swing phase at a greater pace than the HJC-FF to achieve minimal foot clearance [35], thus presenting a higher ratio of change. Abnormal gait deviations leading to changes in DLLs, such as increased knee flexion during the stance phase, might induce short DLLs or stiff knee gait during the swing phase which could cause elongation of the DLLs, demonstrating abnormal changes in the leg length versus normal ratio. In addition, HJC-FF might perform differently owing to its dependency on ankle joint movements, thereby equine gait or drop foot during the swing phase might lead to an altered HJC-FF pattern and change in ratio. Thus, DLLs are dependent on the amount and timing of gait deviation. A higher discrepancy in HJC-FF might indicate ankle joint involvement. Patients presenting with a pathological gait have been found to exhibit different DLL patterns and discrepancies [20], which accordingly should lead to a different maximal to minimal DLL ratio, differentiating from normal gait pattern. DLLs were normalized to the physical measurement of the ASIS to MM in order to formulate a reference for abnormal gait, allowing the identification of abnormal changes in DLL patterns as well as a discrepancy in the magnitude of DLLs due to gait deviations.

Limitations of our study may be related to the fact that the normative dataset was based on young healthy participants assumed to present normal kinematics when walking at their convenient walking speed. Age, body characteristics, and walking speed might cause changes in kinematics and influence DLLs. A wider spectrum of normal healthy subjects should serve as a normal database comparing a matched pathological gait. However, DLL discrepancy should not be affected unless discrepancies occur in gait kinematics. In addition, DLLs patterns, discrepancies, and ratios between maximal and minimal DLLs during the gait cycle should be measured amongst patients presenting with a pathological gait in order to evaluate the ability of this measurement to discriminate between normal and abnormal gait and correlate between gait deviations and the different DLLs.

## 5. Conclusions

DLL measured during the gait cycle based on a valid and reliable gait model, performed on 40 healthy subjects, was found to be consistent and trustworthy. Normal values were set for DLL including patterns of change during the gait cycle, time of maximal and minimal DLLs, and ratio of change. DLLs were also normalized and might serve as a reference for abnormal gait deviation reflected by abnormal DLLs, which is beyond the scope of the study. Abnormal DLLs might indicate abnormal gait deviations and might promote a new perspective of analyzing gait alongside kinematic and kinetic data, which might consequently improve treatment decision-making. However, this was not evaluated in the present study, and further studies measuring the effect of abnormal gait on DLLs are needed to validate this measurement.

## Data Availability

For availability of data and materials, please contact correspondence author for data requests.
